# Reply to Al Ebrahim, K.E. Comment on “Leivaditis et al. Between Air and Artery: A History of Cardiopulmonary Bypass and the Rise of Modern Cardiac Surgery. *J. Cardiovasc. Dev. Dis*. 2025, *12*, 365”

**DOI:** 10.3390/jcdd12100411

**Published:** 2025-10-17

**Authors:** Vasileios Leivaditis, Andreas Maniatopoulos, Francesk Mulita, Paraskevi Katsakiori, Nikolaos G. Baikoussis, Sofoklis Mitsos, Elias Liolis, Vasiliki Garantzioti, Konstantinos Tasios, Panagiotis Leventis, Nikolaos Kornaros, Andreas Antzoulas, Dimitrios Litsas, Levan Tchabashvili, Konstantinos Nikolakopoulos, Manfred Dahm

**Affiliations:** 1 Department of Cardiothoracic and Vascular Surgery, Westpfalz Klinikum, 67655 Kaiserslautern, Germany; vnleivaditis@gmail.com (V.L.); mdahm@westpfalz-klinikum.de (M.D.); 2 Department of Electrical and Computer Engineering, Democritus University of Thrace, 67100 Xanthi, Greece; amaniatopoulos@gmail.com; 3 Department of General Surgery, General Hospital of Eastern Achaia–Unit of Aigio, 25100 Aigio, Greece; vkatsak@gmail.com (P.K.); levedis6@gmail.com (P.L.); kornaros.nikolaos@hotmail.com (N.K.); tchabashvili.alexander@gmail.com (L.T.); 4 Department of Cardiac Surgery, Ippokrateio General Hospital of Athens, 11527 Athens, Greece; 5 Department of Thoracic Surgery, School of Medicine, Attikon University Hospital, National and Kapodistrian University of Athens, 12462 Athens, Greece; sophocmit@yahoo.gr; 6 Department of Oncology, General University Hospital of Patras, 26504 Patras, Greece; lioliselias@yahoo.gr; 7 Department of Surgery, General University Hospital of Patras, 26504 Patras, Greece; vigarant@yahoo.com (V.G.); kostastasiosmd@gmail.com (K.T.); a.antzoulas@hotmail.com (A.A.); 8 John Radcliffe Hospital Emergency Department, University Hospitals NHS Foundation Trust, Headley Way, Headington, Oxford OX3 9DU, UK; 9 Department of Surgery, General Hospital of Lamia, 35100 Lamia, Greece; dimlitsas@icloud.com; 10 Department of Vascular Surgery, General University Hospital of Patras, 26505 Patras, Greece; konstantinosn@yahoo.com

We are grateful to Dr. Al Ebrahim for his thoughtful and constructive commentary on our historical review of cardiopulmonary bypass (CPB) [1,2]. His remarks highlight a crucial aspect of medical history that deserves greater attention—the intellectual contributions of Arab and Islamic scholars to the early understanding of cardiopulmonary physiology, which underpins the eventual development of CPB [1].

Our review traced the journey from early physiological hypotheses to the technological triumph of the heart–lung machine in the mid-20th century. However, we fully acknowledge that the scientific lineage of CPB extends far deeper into antiquity and the medieval period. The recognition of Ibn al-Nafīs (1210–1288) as the first to describe pulmonary circulation correctly is particularly significant. In his Commentary on Avicenna’s Canon (c. 1242), Ibn al-Nafīs boldly rejected Galen’s concept of invisible interventricular pores and explained that blood flows from the right ventricle to the lungs via the pulmonary artery, mixes with air in the spongy pulmonary tissue, and returns to the left ventricle through the pulmonary vein [3]. This paradigm-shifting observation anticipated by centuries the principles later confirmed by William Harvey’s description of systemic circulation.

Equally noteworthy is the intellectual milieu that enabled such progress. Scholars such as Avicenna (Ibn Sīnā, 980–1037), Ibn Rushd (Averroes, 1126–1198), and Ibn Zuhr (Avenzoar, 1091–1161) not only preserved and systematized Greco-Roman medical knowledge but also challenged prevailing dogmas, encouraging empirical observation and critical reasoning [4]. Their work created fertile ground for Ibn al-Nafīs to question Galenic orthodoxy, illustrating that advances in science often arise from the cross-pollination of cultures and disciplines.

The story of CPB therefore reflects more than a technological breakthrough of the 20th century; it exemplifies the cumulative and multicultural nature of medical discovery. By highlighting these historical layers, we celebrate the continuity of human curiosity and innovation that made modern cardiac surgery possible. Such acknowledgment is not only historically just but also relevant today, as contemporary medicine increasingly relies on international and interdisciplinary collaboration.

We also appreciate Dr. Al Ebrahim’s observations on the continued evolution of CPB technology. Over the past seven decades, significant refinements, such as biocompatible circuits, heparin-bonded tubing, miniaturized extracorporeal circulation (MiECC) systems, membrane oxygenators, and most recently artificial intelligence-assisted perfusion, have markedly enhanced the safety and efficiency of CPB [2,5]. These innovations have minimized systemic inflammatory responses, improved hemodynamic control, and expanded the range of procedures safely performed with extracorporeal support. Despite the rise in off-pump coronary surgery and percutaneous interventions, CPB remains indispensable for complex congenital, valvular, and ischemic heart procedures [6]. Its continued refinement exemplifies how historical innovations can evolve without losing relevance.

Finally, we believe that recognizing historical contributions, particularly those from diverse cultural contexts, strengthens our understanding of CPB’s legacy and provides a broader narrative of progress in cardiac surgery. It serves as a reminder that future advances, such as the integration of computational modeling, robotics, and machine-learning-driven perfusion strategies, will likewise depend on the collaboration of engineers, clinicians, ethicists, and innovators from around the world.

In conclusion, we thank Dr. Al Ebrahim for bringing deserved attention to the Arab–Islamic scholars whose early insights into cardiopulmonary physiology paved the way for one of the greatest achievements in modern surgical history. Their contributions remind us that medical progress is a tapestry woven by many cultures and generations—a legacy that continues to inspire and guide innovation in cardiac surgery today.
